# Barriers & facilitators to healthcare and social services among undocumented Latino(a)/Latinx immigrant clients: Perspectives from frontline service providers in Southeast Michigan

**DOI:** 10.1371/journal.pone.0233839

**Published:** 2020-06-05

**Authors:** Monika Doshi, William D. Lopez, Hannah Mesa, Richard Bryce, Ellen Rabinowitz, Raymond Rion, Paul J. Fleming

**Affiliations:** 1 Department of Health Behavior and Health Education, University of Michigan School of Public Health, Ann Arbor, MI, United States of America; 2 Community Health and Social Services (CHASS) Center, Detroit, MI, United States of America; 3 Washtenaw Health Plan, Ypsilanti, MI, United States of America; 4 Packard Health, Ann Arbor, MI, United States of America; University of Montreal, CANADA

## Abstract

Immigration- and enforcement-related policies and laws have significantly and negatively impacted the health and well-being of undocumented immigrants. We examine barriers and facilitators to healthcare and social services among undocumented Latino(a)/Latinx immigrants specifically in the post 2016 US presidential election socio-political climate. By grounding our study on the perspectives of frontline providers, we explore their challenges in meeting the needs of their undocumented clients. These include client access to healthcare and social services, the barriers providers face in providing timely and effective services, and avenues to reduce or overcome factors that impede service provision to improve quality of care for this population. Data are from 28 in-depth interviews with frontline healthcare and social service providers. Based on data analysis, we found that the domains of the Three Delays Model used in obstetric care provided a good framework for organizing and framing the responses. Our findings suggest that these undocumented clients encounter three phases of delay: delay in the decision to seek care, delay in identifying and traveling to healthcare facilities, and delay in receiving adequate and appropriate care at healthcare facilities. Given the current socio-political climate for immigrants, healthcare and social services organizations that serve undocumented clients should adapt existing services or introduce new services, including those that are not site-based.

## Introduction

Over the last two decades, immigration- and enforcement- related omnibus laws are among the most significant policies related to the health and overall well-being of undocumented immigrants in the United States, the vast majority of whom have origins in Latin America [1]. Increasingly, the federal government has shifted responsibility of immigration policy enforcement to states and municipalities through initiatives such as the Secure Communities Program, the Priority Enforcement Program, and Section 287(g) [2,3]. Additionally, some states and municipalities have passed restrictive laws and local ordinances targeting undocumented immigrants [4]. Emerging research suggests that these federal- and state- level immigration- and enforcement-related policies and laws may negatively impact the health and overall wellbeing of immigrants, particularly those who are undocumented, and US-born co-ethnics. Rhodes et al., for example, found that Latina mothers in North Carolina, independent of documentation status, sought prenatal care later and received lower-quality care than non-Latina mothers after local police took responsibility for enforcing federal immigration laws in agreement with Immigration and Customs Enforcement (ICE)–a federal law enforcement agency primarily responsible for immigration and customs enforcement [5]. Furthermore, participants reported mistrust and avoidance of health services, sacrificing their health and the health of their family members. In another study, participants reported higher rates of immigration enforcement stress and lower self-rated health following an immigration raid in Washtenaw County, Michigan [6]. The potentially detrimental health effects of immigration- and enforcement-related omnibus laws on undocumented immigrants are exacerbated by policies related to labor and employment, education, and driver’s licenses. Restricted access to labor markets and experiences with unjust and unfair working conditions have been shown to not only reduce material well-being but also to exacerbate feelings of exclusion and isolation [7]. Legislation that restricts access to education for undocumented immigrants may also have detrimental consequences for social and economic mobility, as well as deleterious long-term health effects, by hindering access to opportunities and resources that promote positive downstream health outcomes [8]. Regulatory policies related to driver’s licenses, which often make it impossible for undocumented immigrants to drive legally, have been found to affect physical and social mobility, including ability to access healthcare and social services [9].

Social and economic policies are also health policies when assessed within the context of social determinants of health [10]. As forms of structural racism, these policies characterize the surround, the features of settings in everyday life [11], that limits access to healthcare and social services for undocumented immigrants and directly impacts fundamental causes of disease by shaping access to life opportunities that influence health [12]. Furthermore, restrictive and regulatory economic and social policies can negatively influence individual health behaviors. In past empirical studies, for example, researchers have overwhelmingly found that fear of deportation is a significant factor in influencing access to and utilization of healthcare services among undocumented immigrants [13–15], signaling potentially high levels of unmet health needs [5,16–17]. Moreover, the reach of restricting economic and social policies has been found to extend to and negatively influence interpersonal interactions. In their study, Rhodes et al. found differential treatment of Latina patients by healthcare providers following implementation of immigration laws and/or enforcement [5]. The resulting mistrust from overt and/or perceived experiences with discrimination can potentially anchor a vicious cycle that negatively influences health seeking behaviors, as well as emotional and physical health, among undocumented immigrants.

In considering the evidence from emerging research, immigration- and immigrant- related social and economic policies are central to characterizing the surround that influences differential access to and utilization of healthcare services among undocumented immigrants. Furthermore, their health limiting effects cascade beyond individual- and inter-personal levels, to impact the modest number of health provision institutions where undocumented immigrants access healthcare. Except for emergency medical care, undocumented immigrants are ineligible for federally funded public health insurance programs, such as Medicare, Medicaid, and the US Affordable Care Act. Through use of their own funds, some states and local governments offer healthcare coverage to undocumented immigrants. However, barring any obstacles, ‘safety-net’ providers, such as public and not-for-profit hospitals, migrant health centers, and federally qualified health centers (FQHCs)–community-based health care providers that are funded through the United States’ Health Resources and Services Administration to provide primary and behavioral healthcare services in underserved areas–are often central points of access among undocumented immigrants seeking healthcare and social services. Thus, perceived or actual barriers to healthcare and social services within these limited number of provision sites can further compound existing challenges, exacerbate the level of unmet needs among undocumented immigrants, and potentially limit providers’ ability to deliver timely and effective care.

Our study is the first to examine barriers and facilitators to healthcare and social services among undocumented Latino(a)/Latinx immigrants in Southeast Michigan from the perspectives of frontline service providers. There are an estimated 130,000 undocumented immigrants in Michigan [18]. Restrictive policies related to driver’s licenses, insurance coverage, as well as labor market and working conditions characterize the state-level context [19–20]. Furthermore, our study implementation sites, Washtenaw County and Detroit, are in a “Constitution Free Zone”–a zone where protection from random and arbitrary stops and searches does not *fully* apply within 100-miles from the US-Canadian border and where border patrol agents have certain additional authorities such as conducting “routine searches” without a warrant or even suspicion and operating immigration checkpoints [21]. While specific to Michigan, these broad characteristics are not dissimilar to other regions/states in the United States.

In this paper, we explore frontline service providers’ perceptions of the challenges faced by their undocumented Latino(a)/Latinx clients in accessing healthcare and social services. Further, we explore these perspectives to examine the barriers they themselves face in providing timely and effective services, and we solicit their thoughts on avenues to reduce or overcome those factors that impede healthcare and social service provision to ultimately improve quality of care for this population. We approach our exploration by ascertaining insights and experiences of frontline service providers affiliated with institutions (i.e., two FQHCs and one non-profit organization in Southeast Michigan) where undocumented immigrants access services. Moreover, unlike past studies, our examination is grounded within the context of the post 2016 US presidential election socio-political climate, one characterized by an intensification of rhetoric, policies and laws against immigrants generally and undocumented immigrants more specifically. Furthermore, our analysis revealed that undocumented clients potentially encounter three phases of delay, leading us to use Thaddeus and Maine’s model [22] to organize and frame our findings. Therefore, we applied the Three Delays Model [22] post hoc to (a) contextualize barriers to healthcare and social services among undocumented Latino(a)/Latinx clients from the perspectives of frontline service providers, (b) explore relationships between barriers, and (c) identify/unpack/appraise strategies towards effective interventions to reduce or overcome barriers, ultimately promoting timely and effective provision of healthcare and social services and improving quality of care for this population.

### Guiding framework: The three delays model

**Fig 1:**
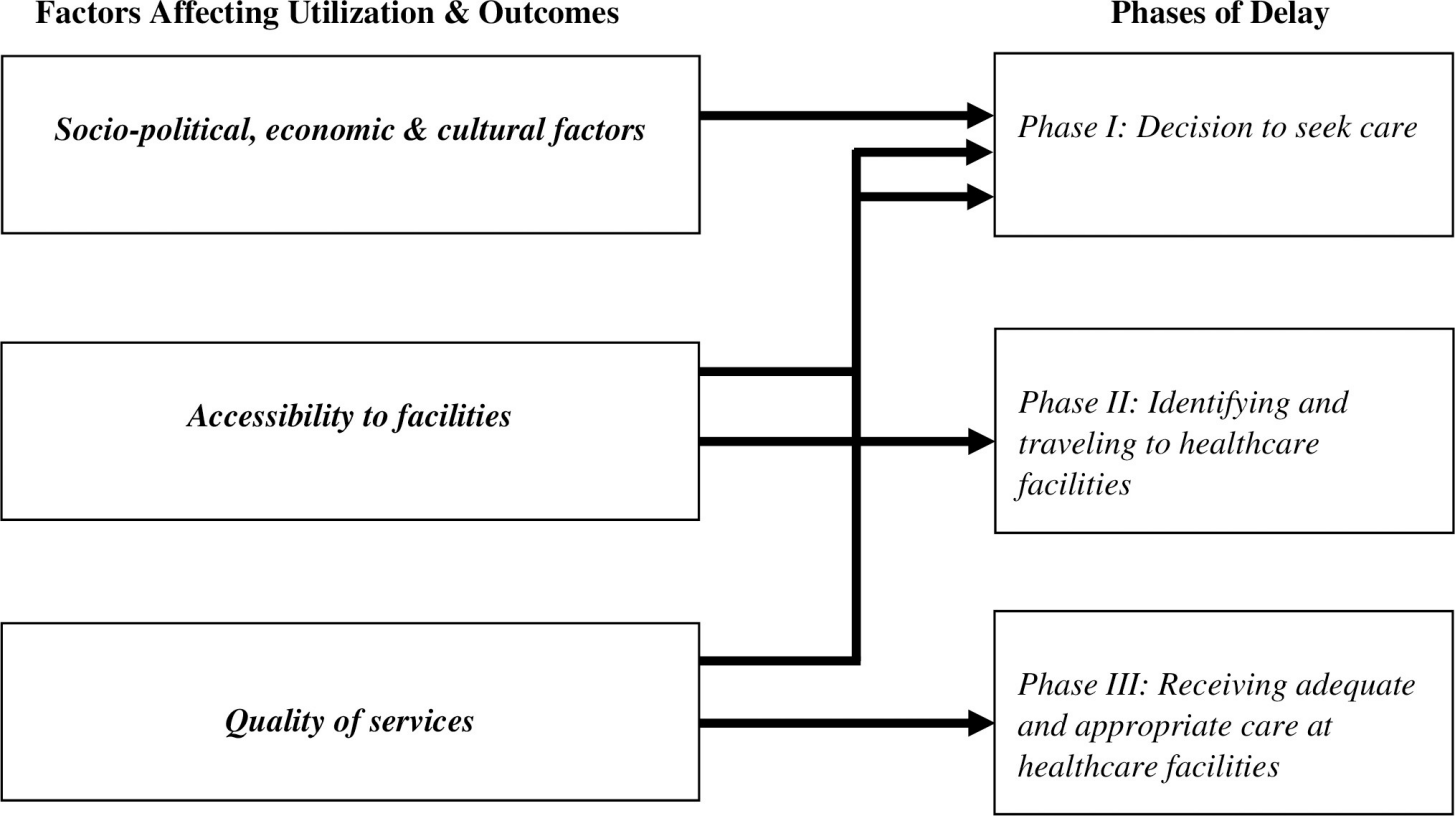
Three delays model.

The Three Delays Model, conceived by Thaddeus and Maine, has traditionally been used to “identify barriers to the provision and utilization of high quality, timely obstetric care” (p. 1092) [22]. In their conceptual framework, (Fig 1), Thaddeus and Maine outline three phases of delay that contribute to preventing maternal mortality [22]:

- Phase I: Delay in the decision to seek care
- Phase II: Delay in identifying and traveling to healthcare facilities
- Phase III: Delay in receiving adequate and appropriate care at healthcare facilities

Each phase of delay is influenced by a set of factors affecting utilization and outcomes. Socio-political, economic, and cultural factors shape the delay in the decision to seek care. Examples include social context, financial costs, and individual decision-making. Factors associated with accessibility to facilities influence the delay in identifying and traveling to healthcare facilities. Examples include feasibility and availability of transportation and knowledge of the healthcare system. The delay in receiving adequate and appropriate care at the healthcare facilities is determined by factors associated with the quality of services in facilities. Examples of relevant factors include facility environment, including capacity and competency of healthcare personnel. Furthermore, as depicted in Fig 1, the decision to seek care (Phase I delay) is not only shaped by socio-political, economic, and cultural factors, but also influenced by factors associated with accessibility to facilities (affect Phase II delay) and factors associated with perceived quality of services in facilities (affect Phase III delay).

This conceptual framework has been widely used across settings such as Malawi, Mozambique, and Indonesia to understand and address the multiple factors that contribute to maternal mortality [23–25] adapted to address neonatal and child morality in India, Tanzania, Sudan, and Uganda [26–29], and to examine barriers to civil registration in Indonesia [30]. Guided by our analysis, which revealed that undocumented clients potentially encounter three phases of delay, we believe that this integrated conceptual framework could be applied post hoc to our data to organize and frame our findings, and that it can also be useful for identifying feedback effects between barriers; that is, for determining relationships between barriers and their potential to compound one another. Thus, we use the Three Delays Model to present our analysis of uptake and utilization of healthcare and social services among undocumented immigrants as shared by frontline service providers and to communicate our findings. Moreover, by better pinpointing where barriers exist, we can more effectively identify/unpack/appraise intervention strategies to address those barriers to ultimately promote timely and effective provision of healthcare and social services and improve quality of care for this population.

## Methods

This study is part of a larger community-academic research collaboration between healthcare and social service organizations in Southeast Michigan, including a FQHC in Detroit, a FQHC in Washtenaw County, and a non-profit organization co-located with the county health department also in Washtenaw County, and the University of Michigan. Grounded in the principles of community-based participatory research [31–32], the research collaboration/project was guided by our partners’ intentions to better meet the needs of their immigrant clients. Informed by their ongoing work and daily interactions with community members, our collaborators suggested downward shifts in uptake and utilization of healthcare and social services among their immigrant clients following the 2016 presidential election. The larger project seeks to unpack the context, expand understanding through engagement of all actors (e.g., service providers and clients) and inform modification of existing services accordingly to better serve immigrant clients. While we currently focus on perspectives of service providers, the next phase of our work will center on perspectives and experiences of clients.

For this phase of the research, we focused on healthcare and social service providers at three partner sites in Southeast Michigan, namely a FQHC in Detroit, a FQHC in Washtenaw County, and a non-profit organization co-located with the county health department also in Washtenaw County. Both Detroit and Washtenaw County are home to considerably large immigrant populations in the state, including persons with origins in Latin America. The Detroit FQHC, for example, is located in a predominantly Latinx neighborhood and provides healthcare and social services to many neighborhood residents, as well as Spanish-speaking residents from surrounding suburbs. In contrast, the Washtenaw FQHC is located in a less urban setting and provides healthcare and social services to a more ethnically and racially diverse population, with a vast majority of the center’s undocumented clients having origins in Latin America. The non-profit organization in Washtenaw County (henceforth: Washtenaw County non-profit), as the Detroit and Washtenaw FQHCs, engages closely with undocumented Latino(a)/Latinx immigrants through its focus on enrolling all county residents in healthcare, including a state-based safety net health insurance program. Therefore, in our study, experiences and perspectives of the participants are grounded within the context of their undocumented clients with origins in Latin America.

### Study design, sampling & recruitment

We utilized semi-structured qualitative interviews in this phase of the research. The interview guide was developed in close consultation with our partners. Working closely with the leadership at each organization, we employed convenience sampling to recruit a diverse group of participants, as defined by their varied roles at the respective study sites, into the study. At each site, the University of Michigan research team identified staff roles within the organization that, as a result of provider-client relationships, could potentially offer intimate insight into the experiences of their immigrant clients and relate the barriers they face in providing timely and effective healthcare and social services to them. Informed by the list of roles, leadership at each site identified and requested participation of staff who had extensive experience working with immigrant communities while also making it clear that their participation was not mandatory. Identified staff were then referred to members of the research team conducting the interviews. Following introductions, members of the research team met privately with each potential participant, explained the study in detail, and gave assurance that participation was voluntary and that the decision to participate/not participate would not affect employment. Moreover, the research staff reiterated that any information shared with their employer and/or colleagues, including the decision to not participate, would be kept anonymous.

We conducted individual in-depth interviews (IDIs) with a cross-section of healthcare and social service providers at each partner site between April 2018 and August 2018. All participants were 18 years of age or older and provided informed consent. A total of 28 healthcare and social service providers were interviewed from the Detroit FQHC (N = 13), the Washtenaw FQHC (N = 5), Washtenaw County non-profit (N = 10).

### Data collection & analysis

We collected information directly from the participants through individual in-depth interviews using a semi-structured interview guide containing mainly open-ended questions and some prompts centered on immigrant clients at their respective organizations. The interviews were conducted by the study’s principal investigator and co-principal investigator, both are PhD level qualitative researchers who have extensive experience working with this population. Moreover, the interviewers were intimately aware of the study questions and met together with other study team members to discuss and determine interview approach prior to the study’s commencement. Furthermore, both interviewers were reflexive and frequently discussed with each other and the study team the ways in which their identities or life experiences could shape the data collection process and analysis. These continuous discussions, which occurred throughout the study period, helped our team minimize how biases impacted our reporting of findings.

The interviews were guided by questions focused on barriers and facilitators to care for their immigrant clients, the impact of the national socio-political climate on their immigrant clients’ ability to access care, challenges to providing timely and effective services, current strategies employed by their organization to address barriers and future recommendations for their organizations, and organizations like theirs, to improve services to immigrant clients. All interviews began with the goal of establishing an understanding of the participant’s role in the organization and ascertaining the demographic profile of the immigrant clients served by their organization. This approach, in turn, promoted rapport building between the participant and the interviewer. All interviews were audio recorded and held in locations that maximized participant confidentiality and safety. Identities of all interviewees were protected through the use of pseudonyms.

Data analysis began during data collection as the interviewers concurrently discussed the themes that were emerging during the interviews. While the post-interview discussions allowed the interviewers to iteratively adapt the interview guide to probe more deeply on emerging themes, they eventually felt they were reaching saturation of key themes related to the research questions after hearing many similar examples and experiences from the interviewees. Interviews were then transcribed verbatim and reviewed by the first (MD), second (WDL), third (HM), and last (PJF) authors, who imported them into NVivo 12 [33] for analysis. We organized the data into topic areas/themes covered by the interview guide. Transcripts were analyzed thematically [34]. Then, we came together in a series of meetings to discuss and develop a codebook with inductive and deductive codes that could help us respond to our research questions. The first author then carried out the analysis using the finalized coding scheme/codebook and applied the Three Delays Model, post hoc, to organize and frame the findings.

After identifying our preliminary results, we presented our findings to the leadership at our partnering organizations for further input and contextualization to ensure appropriate interpretation of the data. We noted no major differences in findings between each site and thus we present the results collectively in the section that follows below.

## Results

### Profile of participants

A total of 28 staff were interviewed from our partner sites: the FQHC in Detroit (N = 13), the FQHC in Washtenaw County (N = 5), and Washtenaw County non-profit (N = 10). Participants represented a cross-section of frontline healthcare and social service providers, including clinical practitioners (N = 5), community health workers/advocates (N = 6), administrators (e.g., directors, managers, coordinators) (N = 5), service representatives/specialists (e.g., enrollment, insurance, benefits) (N = 10) and other (e.g., receptionists, clinic drivers) (N = 2). The vast majority of the participants were female (N = 24), bilingual, English-Spanish speakers (N = 23), and more than half (N = 18) were Latino(a)/Latinx.

### Phase I delay: Decision to seek care

**Fig 2:**
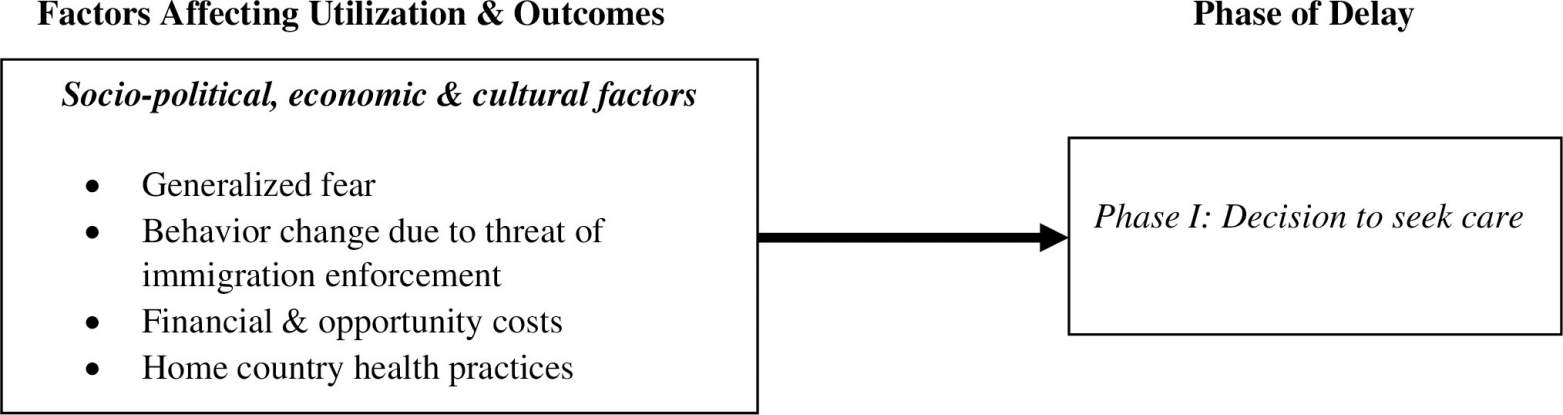
Phase I delay.

Based on our findings, the socio-political, economic and cultural factors that frontline service providers perceived to affect their clients’ decision to seek care, and, in turn, their own ability to provide timely and effective services, include: (a) generalized fear resulting from anti-immigrant rhetoric; (b) behavior change due to threat of immigration enforcement; (c) financial and opportunity costs related to healthcare access; and (d) culturally discordant health seeking practices informed by country of origin (Fig 2).

Overwhelmingly, participants noted that anti-immigrant rhetoric and dehumanizing caricatures, often relayed through the media, as well as a surge in detainments and deportations, characterize the current socio-political context and result in generalized fear, particularly among their undocumented clients. Vanessa at the Washtenaw FQHC described something that her client recently shared with her:

…*whenever you see anything in the media and if you find yourself in that situation, you’re naturally gonna fear*. *So, if all you’re hearing on the news is deportation*, *deportation, deportation it doesn’t matter where you go or what you do*, *there’s gonna be that underlying fear*.

This fear, resulting from the confluence of immigration status (i.e., “not having papers”) and feeling targeted by laws and policies in an ever-increasing restrictive socio-political context, often led undocumented clients to adopt specific immigration-related behaviors, such as self-imposed confinement, as Jennifer noted:

*[Questions clients’ ask themselves] “*…*is it safe to go to the grocery store and get some bread?” “Am I able to go to my doctor’s appointment?” The majority of the families are afraid and so they kind of seclude themselves in their homes*. *They don’t go out. They don’t send their children to school*. *They arrange other ways to get whatever they need*. *And it becomes a very, very, very scary place for lots of people*. *[Jennifer, Washtenaw FQHC]*

Furthermore, participants reported that adoption of protective measures by their undocumented clients, such as home confinement, made it difficult for them to seek care. Linda, for example, shared:

*People are fearful to come out because they’re being stopped, not just in Detroit*, *but the surrounding suburbs where we have a lot of patients coming in*. *Also, because some of them don’t have driver’s licenses, they’re risking [detainment/deportation] if they get caught*. *Some of our patients that are in our project have been deported*. *Some of our patients’ loved ones have been deported*. *Not being able to participate in the activities that they normally would have done, and certainly coming for healthcare is one of them*. *[Linda, Detroit FQHC]*

Thus, fear engendered by anti-immigrant rhetoric, laws, and policies and the related behavior change due to threat of immigration enforcement were reported to be barriers and contributed to a delay in the decision to seek care.

The decision to seek care was shaped by financial costs related to receiving care as well as opportunity costs. The majority of the participants asserted that their clients’ documentation status was directly tied to their healthcare coverage status. For example, Ruben at the Washtenaw County non-profit mentioned: “A lot of them don’t have status or don’t have the right status to get Medicaid.” More specifically, participants attributed gaps in coverage/lack of coverage to the documentation status of their clients and recognized the negative ramifications of this relationship, including prohibitive out-of-pocket costs and the overall lack of options among a group of clients focused on safety, security and self-protection:

*Money, because healthcare is not free here…*. *Unless you are coming with refugee or asylee status you can’t get Medicaid for 5 years except emergency Medicaid*, *so cost is a big barrier*. *[Vanessa, Washtenaw FQHC]*

Along with the prohibitive out-of-pocket expenses directly linked to receiving care, some study participants reported that opportunity costs played an important role in their clients’ decision to seek care. Opportunity costs were reported to include time away from work and the decision to leave children in the care of others while attending to one’s own healthcare needs. Jessica at the Detroit FQHC noted that clients often sacrificed their own health needs when faced with the decision to weigh opportunity costs: “I know a lot of them have young children that were born here. And they feel like ‘[I] cannot do anything that’s gonna leave my children without [me]’.”

Most participants reported that socio-political and economic factors were barriers to accessing healthcare and social services among their clients. A few participants noted cultural factors were potential barriers in clients’ decision to seek care. Those participants largely felt that some clients continued health related behaviors and practices from their country of origin. Lisa at the Washtenaw County non-profit identified the variance in care seeking behaviors by her clients within the context of their mental and physical health related needs:

*There isn’t really a concept of oral health*. *There’s a concept that when you hit your 40s you start pulling your teeth*. *Dental and mental healthcare are treated completely differently*. *People are resistant to access [mental health] cuz sometimes culturally it’s [stigmatized]*.

Furthermore, Mia at the Washtenaw County non-profit indicated that her Latino(a)/Latinx clients’ health seeking behaviors were not preventive but instead were reactive: “Culturally you do not seek out medical attention until you’re like really, really sick.” Although not identified as the primary source of the delay in the decision to seek care, cultural factors were nonetheless reported to contribute to that delay.

Finally, in addition to the socio-political, economic and cultural factors, Phase I delay was also perceived to be shaped by factors associated with accessibility to facilities and quality of services, as discussed in following sections on Phase II and Phase III delays, respectively.

### Phase II delay: Identifying and traveling to healthcare facilities

**Fig 3:**
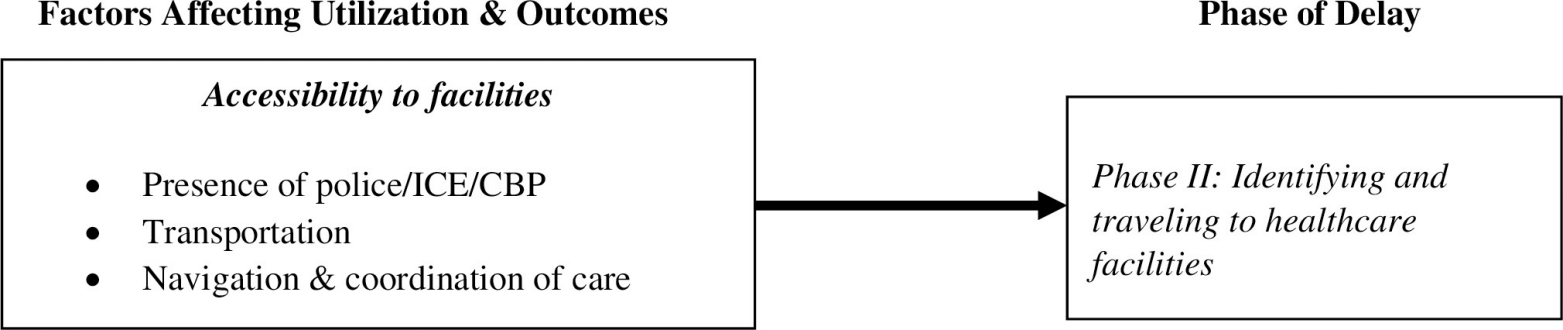
Phase II delay.

Our findings suggest that frontline service providers perceived that their undocumented clients confront a host of factors associated with accessibility to facilities that influence the delay in identifying and traveling to healthcare facilities, and, in turn, their own ability to provide timely and effective services. Clients’ accessibility to facilities plays a dual role in the health-seeking process (Fig 1). It influences their decision-making on whether to seek care (Phase I delay) and it shapes their ability to identify and travel to healthcare facilities (Phase II delay) once the decision to seek care has been made. From the perspectives of frontline staff, factors associated with accessibility to facilities include: (a) presence of local police and/or ICE/Customs and Border Protection (CBP) agents; (b) transportation; and (c) navigation and coordination of care (Fig 3).

Most participants stressed that the presence, as well as the actions, of local police, agents from ICE and/or CBP deterred their undocumented clients from traveling to clinics to receive healthcare and social services.

*I don’t think it’s necessarily ICE*, *I think it is police, particularly the police department*. *They tend to pull people over for minute things like a broken tail light and then if they are feeling particularly aggressive that day, they could contact ICE*. *[Jennifer, Washtenaw FQHC]*

[More fear because of the local police] local police. I mean immigration [ICE] too but local police. They [local police] are like you have a broken tail light, let me see your papers and your documents and things like that. People are afraid of the police. ICE is a **whole** different story. [Mia, Washtenaw County non-profit]

Although the participants varied in their views on whether local police or federal agents posed the greater threat based on the location of the clinic (e.g., proximity to the United States-Canada border; Detroit versus Washtenaw County), an overwhelming majority asserted that both were problematic, posing barriers for their undocumented clients’ accessibility to facilities and subsequently contributing to the delay in identifying and traveling to healthcare facilities. Moreover, the implementation of immigration- and enforcement- related omnibus laws at the local level by police and/or ICE/CBP agents introduced significant barriers for their clients’ ability to travel to healthcare facilities. Consequently, clients were reportedly missing their appointments, as Pablo at the Detroit FQHC shared: “Clients will miss their appointments if they hear that ICE is in the area because they don’t want to be deported.”

Participants also reported a direct link between the presence of local police and/or ICE/CBP agents and their clients’ reluctance to drive, introducing barriers related to transportation and delays with respect to travel to healthcare facilities thereby impeding the provision of timely services.

*I think they’re scared to drive this particular intersection*. *Seems to be almost a weekly or daily occurrence of where people are getting pulled over*. *It’s like a half mile from our clinic so I think that’s kind of a deterrent*. *[Jennifer, Washtenaw FQHC]*

While some undocumented clients were also thought to have experienced transportation issues unrelated to the presence of police and/or federal agents (e.g., not knowing how to drive, not having a car), participants felt that many more were restricted by the presence of police and/or federal agents or the potential threat of their presence especially in the context of driving with expired licenses. For example, T at the Washtenaw County non-profit indicated that regulatory policies related to driver’s licenses made it impossible for their clients to drive legally: “Traveling is a major issue. If people can’t renew their IDs or renew their licenses, that is a big thing.” Thus, participants reported that undocumented clients deferred their healthcare needs out of fear of being targeted while driving, often missing critical health appointments.

*They’re rescheduling [ultrasounds] and everything is because of the immigration status*. *Right now, they’re scared to drive*. *Most of these pregnant ladies have an expired driver’s license for years and they don’t want to risk it*. *They don’t even want to risk coming to the clinic anymore because they don’t have no other transportation but themselves and they’re scared to get out there in the street with an expired driver’s license cuz they hear that the immigration is out a lot more than ever*. *[Laura, Detroit FQHC]*

Moreover, despite transportation assistance from the clinics, Sara at the Detroit FQHC disclosed: “I think transportation is a barrier. And even though we [clinic] have transportation here, I feel like it’s not enough for everyone to get transportation.” Sara’s observation is especially poignant both in the context of supply and demand (high number of clients needing transportation services and the feasibility of serving all) as well as in the related context of immigration raids, with the latter signaling the presence of local police and/or federal agents. For example, following an immigration raid, clients may decide not to utilize an already scheduled transportation service to their appointment, missing their appointment and having to wait not only for the next available appointment but for the time when transportation is also available. Thus, while the provision of transportation services was recognized to be helpful, it did not always seem to meet specific needs.

In addition to the negative effects of structural factors (e.g., presence of local police and/or ICE/CBP agents, implementation of immigration- and enforcement- related policies/laws) at the individual level on the provision of timely and effective services, participants felt that undocumented clients’ perceptions around navigation and coordination of care were potential barriers associated with accessibility to facilities exacerbating their ability to serve their clients. Moreover, participants felt that limited knowledge of the healthcare system or perceived or experienced difficulties associated with navigating the healthcare system most likely posed obstacles for their clients, delaying identification and use of a given facility. For example, Lisa at the Washtenaw County non-profit noted: “You come from a place like the Congo or Romania… or Mexico, [or] if you’re coming from the mountains of Guatemala, it’s like you’re dealing with not just the unfamiliarity of the system, but it doesn’t in a lot of ways make sense.” This quotation makes the important point that the US health system is difficult to navigate even for those who have experience with it, implying that challenges would be much greater for someone who is unfamiliar/has never accessed healthcare prior to arriving in the US. Vanessa at the Washtenaw FQHC underscored the issue: “So I have a range all the way on the other end of the spectrum where they just never had access to healthcare where they’re from. I see that a lot in some of my Central American patients I’ve had where they just never had healthcare.” Thus, clients’ limited exposure to a healthcare system in their country of origin was thought to exacerbate challenges with navigating the US health system; however, the architecture of the US healthcare system was identified to pose potential difficulties independent of clients’ prior exposure.

### Phase III delay: Receiving adequate and appropriate care at healthcare facilities

Finally, our findings indicate that despite overcoming the challenges noted above, frontline service providers reported that undocumented clients often face additional obstacles at the healthcare facility itself thereby compromising timeliness, effectiveness, and quality of care and shaping the third phase of delay–receiving adequate and appropriate care at healthcare facilities. As with the factors associated with Phase II delay, the factors associated with this delay also shape the decision to seek care (Fig 1).

**Fig 4:**
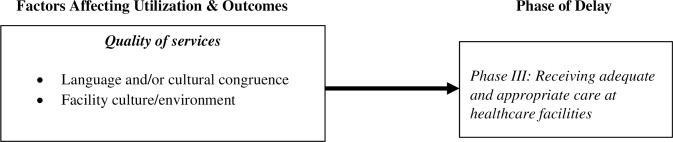
Phase III delay.

Factors associated with quality of services were found to influence the delay in receiving adequate and appropriate care at healthcare facilities. They include: (a) language and/or cultural incongruity between clients and facility staff and (b) facility culture/environment (Fig 4).

Overwhelmingly, participants reported that incongruity in language and/or culture between facility staff and clients often influenced perceptions around and experiences with quality of services, as well as acceptability of services. This subsequently shaped the associated delays–receiving adequate and appropriate care at healthcare facilities as well as the decision to seek care. Mia at the Washtenaw County non-profit captured the potential effects of discordance in language between clients and providers:

*They don’t understand…that’s what prevents them [from accessing services], [the] language*. *Where are the doctors that speak Spanish? It’s very, very disconcerting if you are in pain*, *I mean, if you can imagine that and not be able to tell somebody what the hell is wrong with you*. …*you are trying to explain it and the person is not understanding and this is your health*. *That’s scary*.

In addition to the challenges associated with language and culture incongruency between staff and clients, the participants felt that facility culture/environment influenced experiences with quality of services. More specifically, several participants highlighted discriminatory and racist practices, those that they observed either in their current or past place of employment. Fernanda at the Washtenaw County non-profit recalled an experience from her past place of employment: “I was in my office and I overheard the receptionist saying, yelling to a patient that they needed to bring someone in English. That she wasn’t going to help them. They [patient] spoke Spanish. They [receptionist] would refuse to help.” Moreover, participants viewed these incidents as direct consequences of the current socio-political climate and recognized their detrimental effects on their undocumented clients and their own ability to provide timely, effective and quality care.

### Facilitators to healthcare & social services

**Fig 5:**
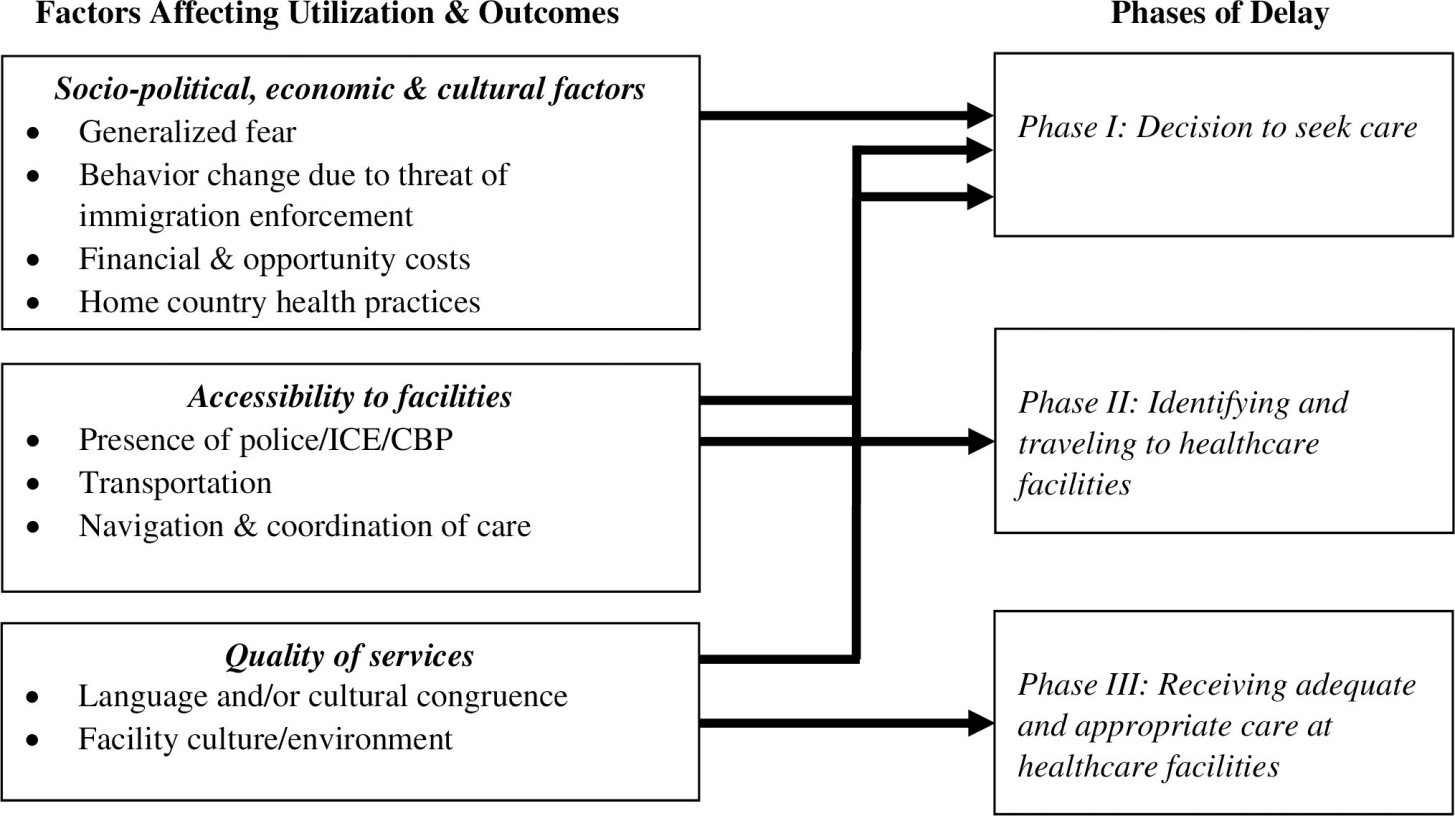
Three delays model (factors detailed).

Our results thus far have focused on factors that pose barriers to healthcare and social services among undocumented clients, those which in turn pose barriers to the provision of timely and effective services by frontline service providers who serve them. However, in our study, we also uncovered current practices at each study site that facilitate access to healthcare and social services among their undocumented clients; practices particularly focused on minimizing the delay in receiving adequate and appropriate care at healthcare facilities by influencing factors associated with quality of services. Moreover, factors associated with the third phase of delay are under the purview and direct control of these facilities. As outlined in the conceptual framework (Figs 1 and 5), factors associated with quality of services also influence clients’ decision to seek care. Thus, the identified facilitative practices have the potential to positively address both the decision to seek care and the receipt of adequate and appropriate care at healthcare facilities.

When asked what leads clients to use their services, the vast majority of participants underscored the importance of establishing/maintaining trust and rapport. Vanessa at the Washtenaw FQHC shared: “I think a lot of it just comes down to just letting them talk, so if you sit and you let them just tell their story…. What I care about is helping.” Moreover, established trust and rapport was reported to result in community endorsement of the site, securing among undocumented clients an informal list of resources that can be trusted. In addition, as Kati at the Washtenaw County non-profit specified, the presence of culturally and linguistically congruent staff was found to be critical to the provision of quality, effective services: “I can just [see] the comfort on people’s faces when they realize that they can actually speak to a Spanish speaker. I mean there’s nothing quite like it.” Further, the provision of quality and affordable care and an open and inviting clinic environment, promoted through campaigns and messaging that signaled “all are welcome here”, were viewed to facilitate timely access among undocumented clients.

While a positive, open, and accepting facility culture and environment were reported to be key in facilitating timely access, participants also asserted that the provision of wrap-around services played a key role. That is, the provision of multiple services within one clinic/site promoted their clients’ ability to efficiently meet manifold needs at once. Lisa at Washtenaw County non-profit captured the ‘one-stop-shop’ model her workplace has attempted to adopt: “We try and we’re trying to meet people where they are at, and sort of provide not completely wraparound services but to wrap more services around.” The participants also reported that they often went above and beyond in their work, providing support and assistance to their clients outside of their specified role and responsibilities (e.g., translating formal communication/letters received from the government). Thus, access to multiple services coupled with the staff’s willingness to go above and beyond with their clients were found to further facilitate and promote trust and ultimately timely access to healthcare and social services. Lastly, participants overwhelmingly echoed the crucial need to meet clients where they are by scheduling off-hour appointments to accommodate working clients.

While current practices by our partners largely focused on factors that minimized the delay in receiving adequate and appropriate care at healthcare facilities, study participants also shared their thoughts and ideas on how to address some of the other structural barriers they previously noted, specifically those that shape the delay in the decision to seek care and the delay in identifying and traveling to healthcare facilities. Participants shared the following ideas but recognized that their ideas needed further exploration through additional research: (a) use of telemedicine to provide clinical healthcare from a distance, (b) re-engagement of practices such as home visits by providers, and (c) harnessing a workforce of community health workers, those with deep connections to the communities served by healthcare facilities, to deliver healthcare and other services to clients’ doorsteps.

## Discussion

In this qualitative study, we examined barriers and facilitators to healthcare and social services among undocumented Latino(a)/Latinx clients from the perspectives of frontline service providers to better understand and address obstacles in their ability to provide timely and effective services. Our exploration is specifically situated in the context of the post 2016 US presidential election socio-political climate, one characterized by an intensification of rhetoric, policies and laws against immigrants generally and undocumented immigrants more specifically. Our analysis revealed that undocumented clients potentially encounter three phases of delay: (a) delay in the decision to seek care (Phase I), (b) delay in identifying and traveling to healthcare facilities (Phase II), (c) delay in receiving adequate and appropriate care at healthcare facilities (Phase III), leading us to use Thaddeus and Maine’s model [22] to organize and frame our findings. Each phase of delay is modified by a set of factors that affect utilization and outcomes, and, moreover, factors associated with Phase II and III delays also affect Phase I delay.

We sought to (a) contextualize barriers to healthcare and social services among undocumented Latino(a)/Latinx clients from the perspectives of frontline service providers, (b) explore relationships between barriers, and (c) identify/unpack/appraise strategies towards effective interventions to reduce or overcome barriers, ultimately promoting timely and effective provision of healthcare and social services and improving quality of care for this population. Our findings suggest that, from the perspectives of frontline service providers, undocumented clients confront a multitude of barriers when accessing healthcare and social services, these that in turn present obstacles in their ability to provide timely and effective services.

The delay in the decision to seek care was largely reported to be shaped by a socio-political context, characterized by anti-immigrant rhetoric as well as a perceived surge in detainments and deportations, that catalyzed fear among undocumented clients. The threat of immigration enforcement engendered changes in behavior (e.g., increased self-confinement) that promoted safety and security but impeded timely access to healthcare and social services, a finding corroborated by prior studies [5, 35–38]. Our study also uncovered the role of opportunity costs and cultural factors in clients’ decision to seek care. Participants stressed that the direct economic burden of paying out of pocket for services, while significant, wasn’t the only consideration in their clients’ decision to seek care. That is, clients often weighed how their decision to seek care might impact their employment (e.g., fear of losing work) and/or their children, specifically the consequences of being detained/deported in the process of seeking care while their children are in the care of others. We also uncovered barriers unrelated to the current socio-political climate. For example, cultural factors were also found to influence this phase of delay, as clients reportedly extended beliefs and practices from their country of origin by deprioritizing non-life threatening or mental health related needs.

The delay in identifying and traveling to healthcare facilities, which negatively affected providers’ ability to deliver timely and effective services, was influenced by factors associated with accessibility to facilities. More specifically, as supported by prior research [13–15], the presence and/or actions of local police and/or ICE/CBP agents and the direct link to fear of detainment/deportation significantly shaped this delay. In addition, barriers unrelated to the current socio-political climate such as perceived challenges or actual experienced difficulties with respect to navigation and coordination of care were also reported to shape accessibility to facilities; a finding that expands on existing literature.

The delay in receiving adequate, appropriate, and effective care at healthcare facilities was affected by factors associated with quality and acceptability of services. That is, the efficiency and effectiveness with which facilities, such as our study sites, provide healthcare and social services to undocumented clients was found not only to be influenced by upstream, macro-level factors such as current US immigration- and immigrant related social and economic policies but also by the intra-organizational environment, such as operating procedures and organizational policies. Within this meso-level context, language and/or cultural incongruence of staff/clinicians and uninviting, insensitive facility environments were reported to be potential barriers to timely and effective services.

Collectively, our findings suggest that barriers to healthcare and social services among undocumented clients are largely exacerbated by the current socio-political climate, one characterized by an intensification of rhetoric, policies and laws against immigrants generally and undocumented immigrants more specifically. In the absence of comprehensive immigration reform, which if realized, has the potential to address the negative effects of structural barriers on access to services, ‘safety-net’ institutions can incorporate or amplify programs that directly address mutable barriers (e.g., transportation, finances, and navigation/coordination of care). For example, the provision of transportation services by facilities could be expanded (e.g., number of vans and drivers) and extended to reach previously un-/under-served local geographic areas. Moreover, facilities can explore alternate avenues to service provision such use of telemedicine, home visits by providers, and the use of community health workers to deliver healthcare and other services to clients’ doorsteps.

Institutions/organizations can also explore additional funding mechanisms to alleviate service-related financial burdens imposed on their undocumented clients. Further, current navigator models (e.g., patient navigator program in cancer care) can be adapted to facilitate navigation and coordination of care among undocumented clients. The aforementioned tactics, however, are resource intensive and may not be easily attainable for many institutions/organizations. In addition to the mutable barriers already mentioned, practices that improve quality of services can minimize delays related to receiving adequate, appropriate, and effective care, as well as the delay in the decision to seek care. As demonstrated by our partnering sites, establishing/maintaining trust and rapport, prioritizing the presence of culturally and linguistically congruent staff, and providing quality, affordable and comprehensive services in an open and inviting environment are specific strategies that can facilitate access, as well as promote and improve timely and effective provision of services among undocumented clients.

## Limitations

Our findings should be understood within the context of some limitations. As a qualitative study, our findings should not be considered as generalizable to all undocumented immigrants but rather that the key themes we present may be transferable to undocumented immigrants in other communities with similar socio-political profiles. Furthermore, while we did feel we reached saturation of key themes, there are certain findings that we were unable to explore in-depth (e.g., the use of telemedicine to overcome barriers). Future research could investigate these findings further and provide a more in-depth analysis. In addition, our findings may not be reflective of the views and experiences of undocumented clients themselves. The client perspectives and experiences will be presented from another phase of our research. However, our study’s goal was to better understand factors that frontline service providers viewed as barriers in their ability to provide timely and effective services to their undocumented clients particularly within the context of the post 2016 US presidential election socio-political climate. Future research should also attempt to broaden understanding by centering the views and experiences of undocumented clients in the context of barriers and facilitators to healthcare and social services. Moreover, large-scale quantitative data collection can help determine how prevalent these themes are within immigrant populations, including those who are undocumented, across the United States.

There are several strengths to this study, including the adaptation of the Three Delays Model [22] to analyze and interpret uptake and utilization of healthcare and social services among undocumented clients. Our study is the first to apply this conceptual framework to research on undocumented immigrants generally and in the context of barriers and facilitators to healthcare and social services more specifically, thereby expanding current understanding and advancing existing scholarship. By applying the Three Delays Model [22] to our analysis post hoc, we have been able to pinpoint where barriers exist, to explore the relationships between them, and to identify/unpack/appraise potential intervention strategies to address them. In addition to this, the participants in our study represented a cross-section of frontline providers with varying degrees of interaction with clients allowing for diverse viewpoints and insights into our collective understanding.

## Conclusions

Guided by our analysis, we applied the Three Delays Model [22] post hoc to interpret uptake and utilization of healthcare and social services among undocumented Latino(a)/Latinx clients from the perspectives of frontline service providers. Further, we explored these perspectives to examine the barriers providers themselves face in providing timely and effective services, and we solicited their thoughts on avenues to reduce or overcome those factors that impede healthcare and social service provision to ultimately improve quality of care for this population. Dividing barriers related to uptake and utilization into three distinct phases offers valuable insights into potential points for intervention. The resulting implications for institutions/organizations that serve undocumented clients can vary as they may lead to adapting existing services or introducing new services. In addition to addressing mutable barriers such as transportation, finance, and facility level factors (e.g., culturally and linguistically congruent care, provision of quality and affordable services), in this period of restrictive immigrant- and immigration- related policies and laws, alternate strategies that do not rely on on-site provision of healthcare and social services should also be considered. These strategies, designed to deliver healthcare and other services to clients’ doorsteps, can include (a) use of telemedicine to provide clinical healthcare from a distance, (b) re-engagement of practices such as home visits by providers, and (c) harnessing a workforce of community health workers. Future intervention research should explore the feasibility, sustainability, and viability of these strategies in promoting and advancing the health of undocumented immigrants.
